# Subolesin vaccination inhibits blood feeding and reproduction of *Haemaphysalis longicornis* in rabbits

**DOI:** 10.1186/s13071-020-04359-w

**Published:** 2020-09-18

**Authors:** Seung-Hun Lee, Jixu Li, Paul Franck Adjou Moumouni, Kiyoshi Okado, Weiqing Zheng, Mingming Liu, Shengwei Ji, Soochong Kim, Rika Umemiya-Shirafuji, Xuenan Xuan

**Affiliations:** 1 National Research Center for Protozoan Diseases, Obihiro University of Agriculture and Veterinary Medicine, Obihiro, Hokkaido 080-8555 Japan; 2 College of Veterinary Medicine, Chungbuk National University, Cheongju, 28644 South Korea

**Keywords:** *Haemaphysalis longicornis*, Subolesin, Vaccine, Tick, Akirin, Tick-borne disease

## Abstract

**Background:**

Ticks can transmit numerous tick-borne pathogens and cause a huge economic loss to the livestock industry. Tick vaccines can contribute to the prevention of tick-borne diseases by inhibiting tick infestation or reproduction. Subolesin is an antigenic molecule proven to be a potential tick vaccine against different tick species and even some tick-borne pathogens. However, its effectivity has not been verified in *Haemaphysalis longicornis*, which is a widely distributed tick species, especially in East Asian countries. Therefore, the purpose of this study was to evaluate the effectivity of subolesin vaccination against *H. longicornis* in a rabbit model.

**Methods:**

*Haemaphysalis longicornis* (Okayama strain, female, adult, parthenogenetic strain) and Japanese white rabbits were used as the model tick and animal, respectively. The whole open reading frame of *H. longicornis* subolesin (HlSu) was identified and expressed as a recombinant protein using *E. coli*. The expression was verified using sodium dodecyl sulfate polyacrylamide gel electrophoresis, and the immunogenicity of rHlSu against anti-*H. longicornis* rabbit serum was confirmed using Western blotting. After vaccination of rHlSu in rabbits, experimental infestation of *H. longicornis* was performed. Variables related to blood-feeding periods, pre-oviposition periods, body weight at engorgement, egg mass, egg mass to body weight ratio, and egg-hatching periods were measured to evaluate the effectiveness of subolesin vaccination.

**Results:**

The whole open reading frame of HlSu was 540 bp, and it was expressed as a recombinant protein. Vaccination with rHlSu stimulated an immune response in rabbits. In the rHlSu-vaccinated group, body weight at engorgement, egg mass, and egg mass to body weight ratio were statistically significantly lower than those in the control group. Besides, egg-hatching periods were extended significantly. Blood-feeding periods and pre-oviposition periods were not different between the two groups. In total, the calculated vaccine efficacy was 37.4%.

**Conclusions:**

Vaccination of rabbits with rHlSu significantly affected the blood-feeding and reproduction in *H. longicornis*. Combined with findings from previous studies, our findings suggest subolesin has the potential to be used as a universal tick vaccine.
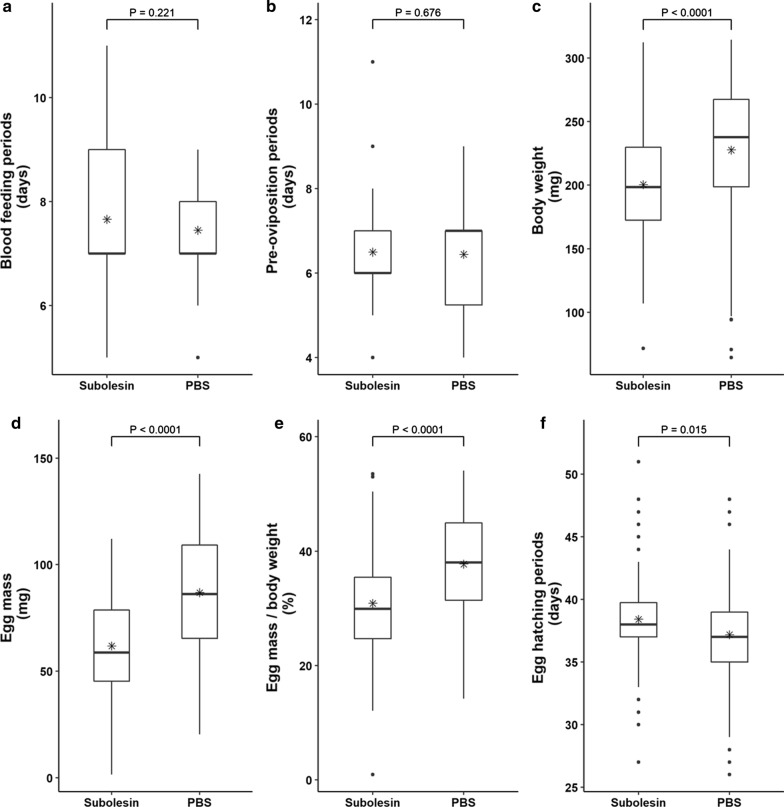

## Background

Ticks are one of the most important vectors and can transmit numerous pathogens covering bacteria, viruses, and parasites including *Anaplasma phagocytophilum*, *Borrelia burgdorferi*, *Babesia* spp., *Theileria* spp., severe fever with thrombocytopenia syndrome (SFTS) virus, and tick-borne encephalitis virus [1]. By transmitting tick-borne diseases, ticks cause huge economic loss to the livestock industry. A heavy tick infestation in animals, even without disease transmission, can cause severe blood loss, resulting in economic problems for breeding farms [2]. Therefore, the control of ticks and tick-borne disease is important in the fields of agriculture and veterinary medicine.

*Haemaphysalis longicornis* is a widely distributed tick species. It is the dominant tick species in Korea and plays an important role in East Asian countries, including China and Japan [3, 4]. Recently, *H. longicornis* invaded North America, raising public attention [5]. Based on recent studies, *H. longicornis* is a proven vector of tick-borne pathogens including SFTS virus and *Babesia gibsoni* [6, 7]. Unfortunately, there are no effective vaccines against both tick-borne pathogens. Tick-borne pathogens can be transmitted transstadially and transovarially [6, 8], which means an infected tick can reproduce to create plentiful infected offspring. Therefore, the prevention of both transstadial and transovarial transmission is important to prevent the spread of tick-borne diseases.

Thus far, there have been efforts to prevent tick-borne diseases in different ways, e.g. using tick-resistant breeds, treatment with acaricides, and development of vaccines [9]. However, widespread treatment with acaricides is limited by factors such as safety concerns, environmental pollution, and the existence of acaricide-resistant tick strains that puts the effectiveness of acaricides in doubt [9]. Regarding vector-borne diseases such as yellow fever, malaria, and theileriosis, many studies have tried to develop vaccines against them; however, a lot of efforts still are required to reach the goal [10–12]. In addition, aiming to control each pathogen entails a huge cost, requires a large workforce and long periods. To overcome these disadvantages, researchers have aimed to develop vaccines against vectors and not for individual pathogens. One of the most well-known tick vaccines is the Bm86-based formulation derived from *Rhipicephalus* (*Boophilus*) *microplus* gut protein [13]. The Bm86-based vaccination is cost-effective and more environmentally friendly than acaricides; however, the Bm86-based vaccines are ineffective against some tick species [14, 15]. In addition, homologs of Bm86 were not effective against other tick species such as *Ixodes ricinus* and *Amblyomma cajennense* [16, 17]. Moreover, the effectiveness of Bm86-based vaccination has not been proven in *Haemaphysalis* spp. [18], which makes it difficult to be used as a universal tick vaccine.

Subolesin is an antigenic molecule and was first identified in *Ixodes scapularis* in 2003 [19]. Subsequent studies revealed that the function of subolesin is related to tick blood-feeding and reproduction using RNAi treatment [20]. Subolesin was initially identified in hard ticks and was subsequently revealed as an ortholog of akirin in insects and vertebrates [21, 22]. Recent studies have shown that this molecule has the potential to be used as a vaccine against different tick species in different hosts. Additionally, the vaccination gives protection against tick-borne pathogens, including *Borrelia burgdorferi*, *Anaplasma marginale* and *Babesia bigemina* [23–25].

Thus far, subolesin has been studied mainly in species of *Ixodes*, *Rhipicephalus* and *Dermacentor* [24, 26]. Regarding *Haemaphysalis* spp., Rahman et al. [27] used RNAi treatment to demonstrate the existence of subolesin in *H. longicornis* and showed that it has an identical function in different tick species. However, the sequence information was defective and not verified in animal experiments. As RNAi treatment and animal experiments do not always show identical results [28, 29], it is essential to evaluate the effectivity of vaccination in animal experiments. Therefore, the purpose of this study was to evaluate the effectivity of subolesin vaccination in *H. longicornis* as a tick vaccine in rabbits.

## Methods

### Ticks and rabbits

In this study, parthenogenetic *H. longicornis* (Okayama strain) was used as the model tick. The tick has been maintained as a colony by routine passage in Japanese white rabbits at the National Research Center for Protozoan Diseases, Obihiro University of Agriculture and Veterinary Medicine, Japan [30]. Four-to-eight-month-old female ticks were used in this study. Before the start of blood-feeding, the ticks were habituated at room temperature with 40% humidity for two weeks. For experimental infestation, female ticks derived from the same batch were used, to reduce the potential bias caused by batch differences.

Japanese white rabbits (specific-pathogen-free animal, female, 9-weeks-old) were purchased from a company (Clea Japan, Tokyo, Japan). The rabbits were housed until 3–8-months-old in a room with a temperature of 25 °C, humidity of 40%, and controlled lighting (i.e. period of light from 6:00 to 19:00 h). The rabbits had access to tap water and commercial pellets (CR-3; CLEA Japan, Tokyo) *ad libitum* throughout the experiments.

### Identification of subolesin in *H. longicornis*

Total RNA was extracted from *H. longicornis* using the Direct-zol RNA Miniprep (Zymo Research, Irvine, CA, USA) after breaking the tick mechanically with liquid nitrogen. cDNA was synthesized using the PrimeScript™ RT-PCR Kit (Takara, Shiga, Japan), according to the manufacturer’s protocols. The extracted cDNA was kept at – 30 °C until further experiments were performed.

Primers were newly designed to amplify the whole open reading frame (ORF) of *H. longicornis subolesin* (HlSu). The primers were designed based on the previously reported sequences of subolesin in other tick species in the GenBank database and cDNA library of *H. longicornis* in our laboratory [31]. Using the primers, polymerase chain reaction (PCR) was performed as per the following cycles: initial denaturation at 94 °C for 5 min, followed by 35 cycles of 94 °C for 30 s, 50 °C for 30 s, 72 °C for 30 s, and a final extension step at 72 °C for 5 min. The primers used in this study are listed in Table 1.

**Table 1:** Primers used in this study

Name	Sequence (5’-3’)^a^	Purpose
HlSu_For	ATGGCTTGTGCGACATTAAAG	Identification and amplification of *H. longicornis* subolesin
HlSu_Rev	TTATGACAAATAGCTTGGAGTGGC	
HlSu_XhoI_For	ctcgagATGGCTTGTGCGACATTAAA	Insertion of restriction enzyme site
HlSu_EcoRI_Rev	gaattcTTATGACAAATAGCTTGGAGTGGC	

^a^Restriction enzyme sites are in lower case

### Cloning, sequencing, and phylogenetic analysis of HlSu

The amplified PCR product was ligated to pGEM-T Easy Vector (Promega, Tokyo, Japan) and was transformed into *Escherichia coli* DH5α. It was cultured on a Luria-Bertani agar plate containing 100 μg/ml ampicillin at 37 °C. Finally, five colonies were selected and cultured for plasmid extraction and sequencing. The extracted plasmids were sequenced using the BigDye Terminator v3.1 Cycle Sequencing Kit (Applied Biosystems, New York, USA), and the results were analyzed using the ABI PRISM 3100 Genetic Analyzer (Applied Biosystems, Carlsbad, CA) according to the manufacturer’s instructions. All the plasmids were sequenced bidirectionally and were aligned.

The obtained sequence was analyzed by BLAST to compare its identity with that of other subolesin sequences. Phylogenetic analysis was performed to analyze the molecular relationship among subolesin identified in insects. The phylogenetic tree was constructed using MEGA 7.0. using the Maximum-Likelihood method and 500 replications [5].

The sequence and molecular weight of rHlSu were deduced using a plasmid Editor v2.0.61.

### Recombinant HlSu (rHlSu) expression and purification

The whole ORF of HlSu was inserted into a pCold ProsS2 vector (TaKaRa, Japan), which has a His- and ProS2-tag, for expression as a recombinant protein. To insert HlSu into the vector, restriction enzyme sites (*Xho*I for the upstream site and *Eco*RI for the downstream site) were added using PCR with the primers listed in Table 1. The inserted expression vector was transformed into *E. coli* BL21(DE3), and recombinant protein was expressed according to the vector manufacturer’s protocols. In brief, the transformed *E. coli* BL21(DE3) was cultured at 37 °C with vigorous shaking until the optical density at 600 nm reached 0.4, and was cooled to 15 °C for 30 min. After that, induction was initiated with isopropyl β-D-1-thiogalactopyranoside concentration of 1.0 mM and was cultured at 15 °C along with shaking for 24 h.

The cultured *E. coli* was washed with phosphate-buffered saline (PBS) three times and broken up using sonification. The recombinant HlSu was purified using His GraviTrap (GE Healthcare, Tokyo, Japan) according to the manufacturer’s protocols. In brief, the column was equilibrated with 10 ml binding buffer (20 mM sodium phosphate, 500 mM sodium chloride, 20 mM imidazole, pH 7.4), and samples were applied. After washing the column with the binding buffer two times, the protein was eluted with 3 ml of the elution buffer (20 mM sodium phosphate, 500 mM sodium chloride, 500 mM imidazole, pH 7.4).

Only the soluble fraction was used for further experiments. Expression and purification of rHlSu were verified using sodium dodecyl sulfate polyacrylamide gel electrophoresis (SDS-PAGE). SDS-PAGE results were visualized by Coomassie blue staining. The protein concentration was estimated by using Pierce BCA Protein Assay Kit (Thermo Fisher Scientific, Tokyo, Japan).

### Production of anti-*H. longicornis* rabbit serum

To produce anti-*H. longicornis* rabbit serum, a rabbit was experimentally infested with *H. longicornis* using the ear bag method, as previously described [32]. Experimental infestation was performed three times; each time, the rabbit was infested with 40 female ticks (20 ticks for each ear), with two-week intervals between infestations. Two weeks after the last infestation, serum was collected from the rabbit.

### Western blotting

The immune response of rHlSu against *H. longicornis* infestation was confirmed using Western blotting. In brief, 0.03 μg rHlSu was separated using SDS-PAGE and transferred to an Amersham Protran 0.45 nitrocellulose Western blotting membrane (GE Healthcare, Tokyo, Japan). After blocking with TBS containing 0.1% Tween 20 (TBS-T) and 3% skimmed milk, the membranes were probed with anti-*H. longicornis* rabbit serum (described above) or naïve rabbit serum diluted with TBS-T at a ratio of 1:600. HRP-conjugated goat anti-rabbit IgG (H+L) cross-adsorbed secondary antibody (Thermo Fisher Scientific) was used as a secondary antibody diluted with TBS-T at a ratio of 1:5000. Finally, the reaction was visualized using Amersham ECL Prime Western Blotting Detection Reagent (GE Healthcare, Tokyo, Japan). The results were scanned using a VersaDoc™ imaging system (Bio-Rad Laboratories, Tokyo, Japan).

To estimate the immunogenicity of rHlSu after vaccination, Western blotting was similarly performed using rHlSu and rHlSu-immunized rabbit serum (described below), except that serum dilution was done at 1:2000.

### Vaccine trial

To evaluate the efficacy of rHlSu as a tick vaccine, two groups were established (vaccination group and PBS group). Each group included three Japanese white rabbits. Before vaccination, the purified rHlSu protein was dialyzed with PBS using Pierce Protein Concentrator 30K MWCO (Thermo Fisher Scientific) according to the manufacturer’s recommendation. In brief, rHlSu in the elution buffer was mixed with 3 ml of PBS and was centrifuged at 8000× *g* leaving a residual 1 ml buffer. Again, the remaining buffer was centrifuged in the same way with 5 ml of PBS.

rHlSu or PBS was then conjugated with adjuvant (TiterMax Gold, Sigma-Aldrich, Japan). In total, 500 µg of rHlSu (or the same amount of PBS for the control group) was mixed with 500 µl of the adjuvant and was intradermally injected into the back of each rabbit. The dose was determined according to the manufacturer’s instructions and based on the body weight of rabbits. The vaccination was boosted twice at two-week intervals. Before each vaccination, blood was collected from the ear vein to obtain sera, and antibody levels were evaluated using enzyme-linked immunosorbent assay (ELISA).

### ELISA protocol

Antibody titers in the sera were evaluated using ELISA. In brief, purified rHlSu (0.25 μg per well) in 50 mM carbonate-bicarbonate buffer was used to coat ELISA plates and left overnight at 4 °C. Blocking of the plate was performed with 100 µl of 3% skimmed milk-PBS at 37 °C for one hour. Next, 50 µl sera diluted at a ratio of 1:100 in 3% skimmed milk-PBS were incubated at 37 °C for one hour. After washing the plate with PBS containing 0.1% Tween 20 (PBS-T) six times, 50 µl of HRP-conjugated goat anti-rabbit IgG diluted at a ratio of 1:4000 with 3% skimmed milk-PBS, was added to each well and incubated at 37 °C for one hour. After washing the plate with PBS-T six times, 100 µl of substrate solution (ABTS, Roche, Tokyo, Japan) was added to each well and kept in the dark for one hour. Optical density value was measured three times at 415 nm using a spectrometer (Corona microplate reader MTP-500, Corona Electric, Ibaraki, Japan).

### Effect of vaccination

Three weeks after the last vaccination, experimental infestation with female ticks was performed as described above. After starting the experimental infestation, the condition of ticks and rabbits was checked daily. The ticks that detached abnormally after the start of feeding (detachment without external force, tick’s body weight < 50 mg), were excluded from the data analysis. During the experimental infestation, five and six ticks in the vaccination group and PBS group, respectively, were excluded from the data analysis due to abnormal detachment. After blood-feeding, the fully engorged ticks were incubated in the dark at 25 °C and saturated humidity for oviposition.

The effect of vaccination was evaluated based on the following factors: blood-feeding period (days), pre-oviposition period (days), body weight at engorgement (mg), egg mass at 10 days after oviposition (mg), egg mass to body weight ratio, and egg period (days). Each parameter was estimated daily. In total, vaccine efficacy was calculated by the formula: (1 − reduction ratio of body weight × reduction ratio of egg mass) × 100.

### Statistical analysis

Differences between groups were analyzed using Student’s t-test or the Mann-Whitney test according to the distribution of data using SPSS Statistics for Windows, Version 26.0 (IBM Crop., Armonk, NY, USA). A *P*-value of less than 0.05 was considered statistically significant.

## Results

### Identification and phylogenetic analysis of HlSu

On cloning and sequencing, the whole ORF of HlSu was 540 bp and the sequence length was identical with that of subolesin in *H. flava* and *H. punctata*, but differed from that in *H. elliptica* (528 bp). Based on a BLASTN search, HlSu showed the highest genetic similarity to subolesin in *Haemaphysalis elliptica* at 93.7% (JX193850). Among *Haemaphysalis* spp., the similarity ranged from 90.0% (*H. punctata*, DQ159972) to 93.7% (*H. elliptica*, JX193850). At the amino acid level, it shows the highest similarity with *H. elliptica* at 96.1% (AGI44626, JX193850) by a BLASTP search.

**Fig. 1:**
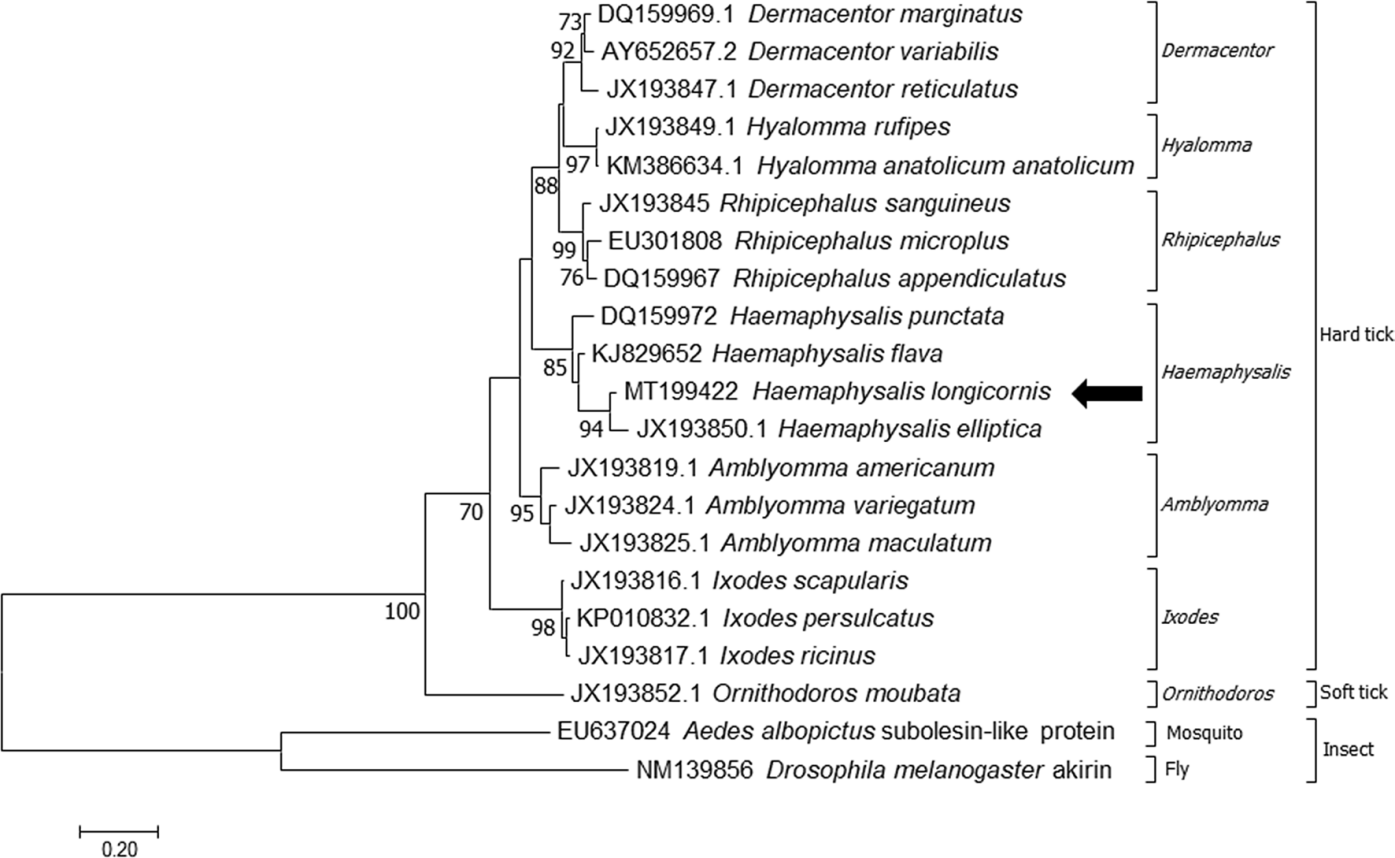
The phylogenetic relationships of *subolesin* identified in this study. For this analysis, other *subolesin* sequences were obtained from the GenBank database. The tree is generated based on the Maximum-Likelihood method with 500 replications using MEGA 7.0. The sequence obtained in this study is indicated by an arrow. Bootstrap values < 70 were omitted

Based on the phylogenetic analysis, subolesin is grouped in a monophyletic clade with other *Haemaphysalis* orthologs including *Dermacentor*, *Hyalomma*, *Rhipicephalus* and *Amblyomma*. Subolesin of *H. longicornis* showed the closest relationship with the subolesin in *H. elliptica*, followed with that of *H. flava* and *H. punctata* (Fig. 1). The obtained sequence was submitted to the GenBank database (accession number: MT199422).

### rHlSu expression, purification, and immunogenicity

**Fig. 2:**
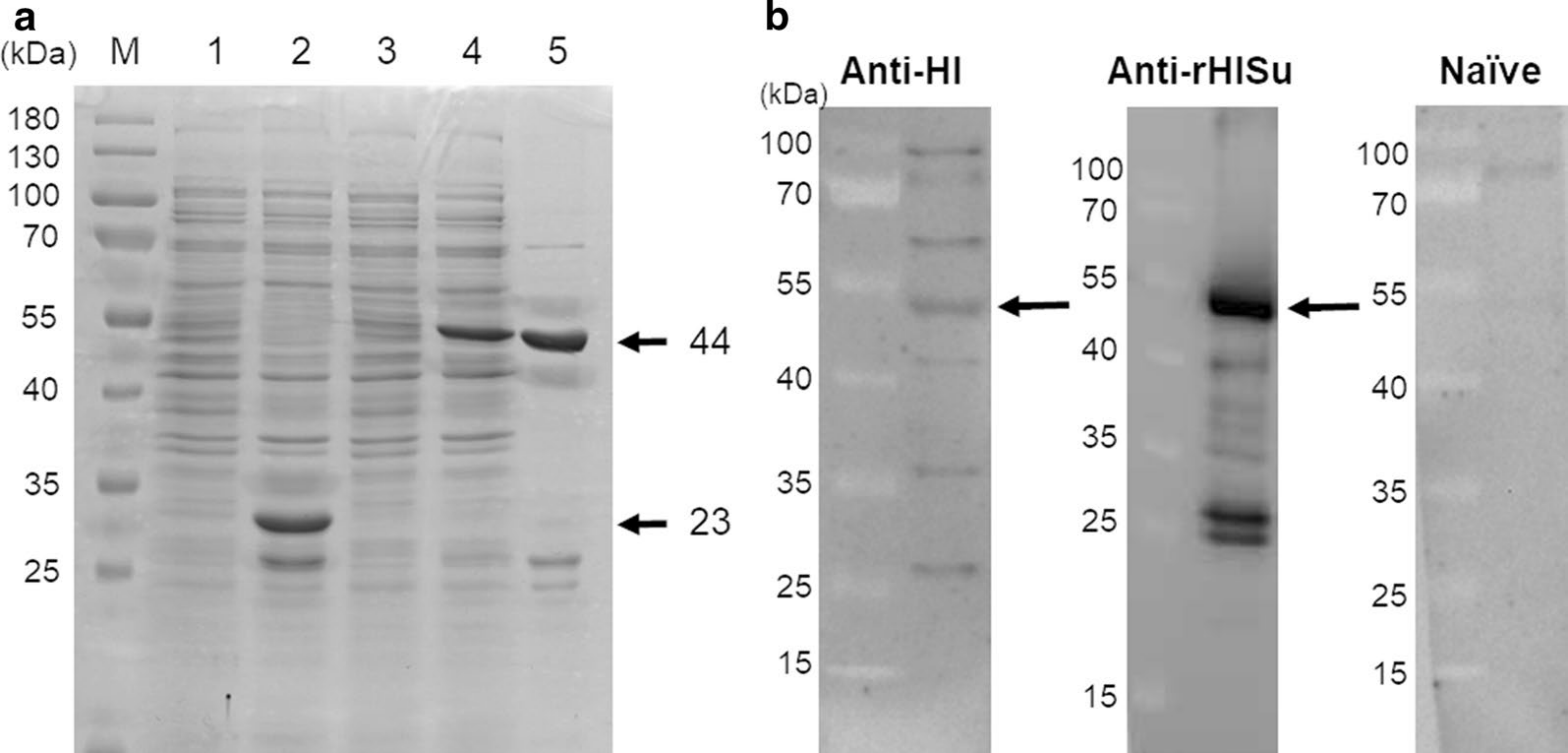
Expression and immune response of rHlSu. **a** Expression and purification of recombinant *Haemaphysalis longicornis* subolesin (rHlSu) using an *E. coli* expression system. Each protein (Lanes 1–4, 6 μg; Lane 5, 2.25 μg) was applied and separated by SDS-PAGE. Lane M: protein marker; Lane 1, empty vector without IPTG induction; Lane 2, empty vector with IPTG induction; Lane 3, vector including subolesin without IPTG induction; Lane 4, vector including subolesin with IPTG induction; Lane 5, purified rHlSu. **b** The immune response of rHlSu was verified using Western blotting. Purified rHlSu (0.03 μg) was used for Western blotting. Serum dilutions were 1:600 for lanes anti-*H. longicornis* serum and Naïve serum, and 1:2000 for lane anti-rHlSu serum. Secondary antibody dilution was 1:5000 for all lanes. Anti-Hl, reaction with anti-*H. longicornis* serum; Anti-rHlSu, reaction with anti-rHlSu serum; Naïve, reaction with naïve serum

The rHlSu was expressed at approximately 44 kDa (tag size, ~ 23 kDa) as expected (Fig. 2a). After purification, the dominant band at the target size remained; thereafter, only purified rHlSu was used in further experiments. Using Western blotting, the immune response of rHlSu was verified (Fig. 2b). At the target size, reactivity was observed in the *H. longicornis*-infested rabbit serum and rHlSu-immunized rabbit serum, but not in naïve serum.

### Vaccination and immune response

**Fig. 3:**
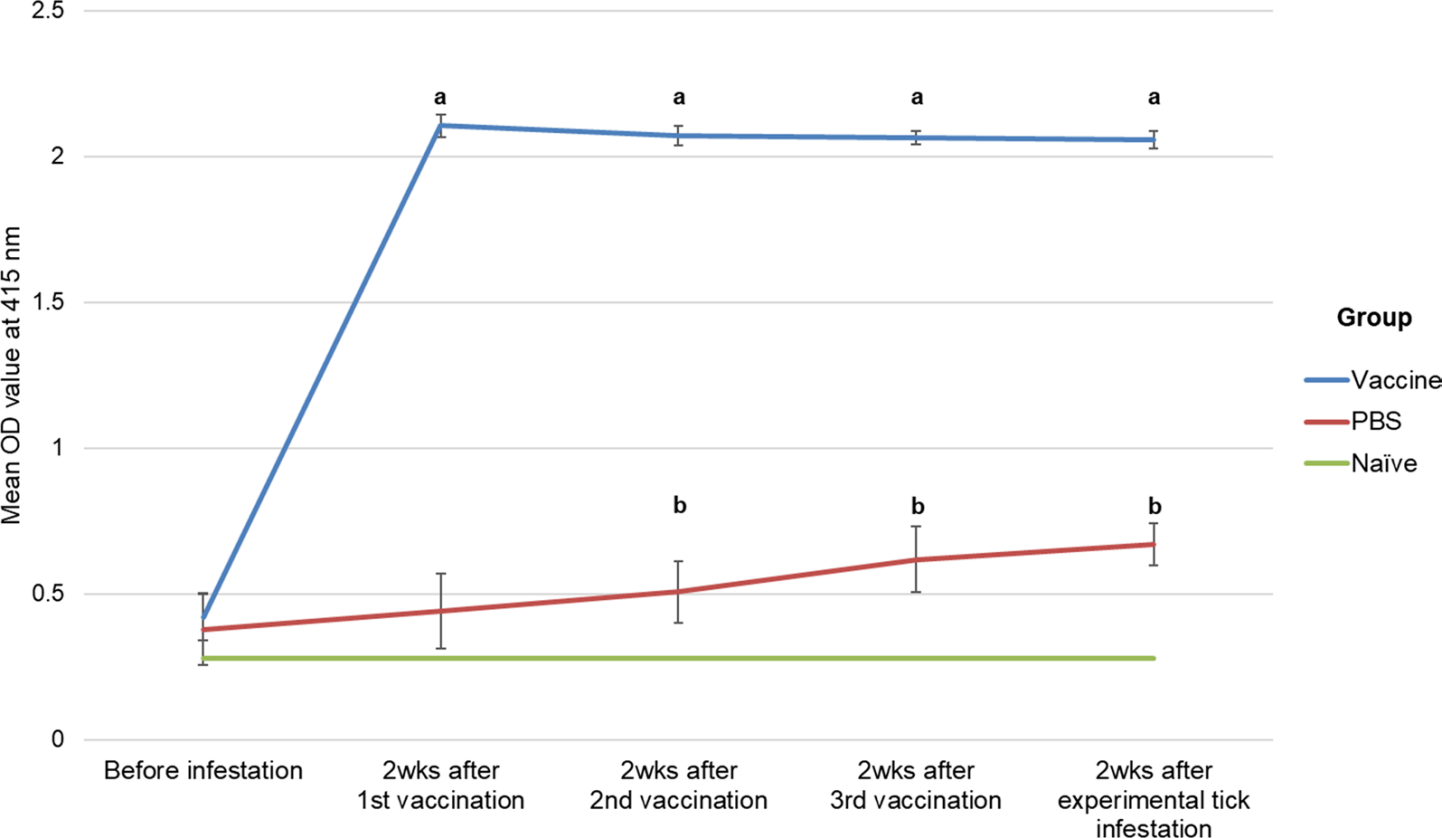
Evaluation of antibody production after vaccination. The vaccination group and PBS group were established and tested. Antibody levels were estimated using ELISA, and the mean antibody titer is shown. Following each vaccination, there was a two-week interval before antibody levels were assessed. Statistical analysis regarding OD values between the two groups was performed using the Mann-Whitney test. **a** *P* < 0.005 to PBS group; **b** *P* < 0.005 to naïve rabbit serum

After the first vaccination, antibody levels in the vaccination group were significantly elevated to three to four times more than that in the PBS group (Mann-Whitney U-test: *U* = 0.000, *Z* = − 3.576, *P* < 0.0001). The antibody levels did not increase further after the second and third vaccination (Fig. 3). Antibody levels in the PBS group did not differ statistically and were elevated when compared to naïve rabbit serum before and after the second immunization, respectively.

### Effect of vaccination against *H. longicornis*

**Fig. 4:**
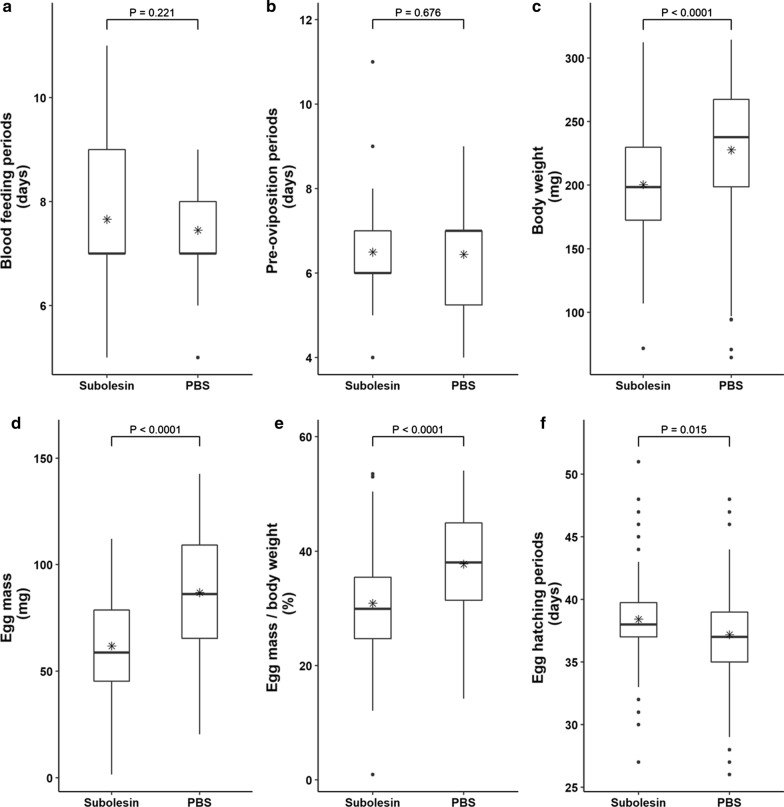
Effect of rHlSu vaccination on rabbits to control *Haemaphysalis longicornis* infestation. The results are shown as box plots. **a** Blood-feeding periods. **b** Pre-oviposition periods. **c** Body weight at engorgement. **d** Egg mass. **e** Egg mass to body weight ratio. **f** Egg-hatching periods. Mean values are indicated with an asterisk. Statistical comparison between the two groups for each parameter was performed using Student’s t-test or Mann-Whitney test

In the rHlSu-vaccinated group, body weight at engorgement (Mann-Whitney U-test: *U* = 4117.000, *Z* = − 4.780, *P* < 0.0001), egg mass at 10 days after oviposition (t-test: *t* = − 7.139, *P* < 0.0001), and egg mass to body weight ratio (t-test: *t* = − 5.840, *P* < 0.0001) were significantly lower than those in the PBS group (Fig. 4; Additional file 1: Table S1). The egg-hatching periods were significantly longer in the vaccinated group than in the PBS group (Mann-Whitney U-test: *U* = 4627.500, *Z* = − 2.432, *P* = 0.015). In cases of body weight and egg mass, the vaccinated group had values that were 12.0% and 28.9% lower than those in the PBS group, respectively. The calculated vaccine efficacy was 37.4%. Blood-feeding periods (Mann-Whitney U-test: *U* = 5922.000, *Z* = − 1.223, *P* = 0.221) and pre-oviposition periods (Mann-Whitney U-test: *U* = 6076.000, *Z* = − 0.417, *P* = 0.676) were not different between the two groups.

## Discussion

Subolesin and its orthologue akirin were identified in different insects, and their molecular information has been analyzed [21, 22]. However, only partial sequence information is available on *H. longicornis* (EU289292), which remains unpublished. As the essential and effective epitope of subolesin has not yet been confirmed [33], this study was initiated to identify and express the whole ORF of HlSu in order to use the entire region as a recombinant protein.

Previous studies have shown that subolesin-based vaccination showed effectivity as a tick vaccine in cattle, rabbits, and mice based on different parameters including body weight, mortality, molting, tick infestation, fertility, and oviposition [22, 25, 26, 34]. Moreover, vaccination also reduced the level of infection with tick-borne pathogens [23–25]. The vaccine efficacy in this study was 37.4%, which was calculated under consideration of the body weight and egg mass. As previous studies were performed under various experimental conditions, such as during the developmental stage; with different tick species, hosts, or adjuvants; and methods to calculate efficacy, it is difficult to directly compare the efficacy of subolesin-based vaccination between studies. In summary, previous studies showed that subolesin-based vaccination protects against tick infestation.

In this study, subolesin vaccination significantly reduced body weight, egg mass, and the ratio of egg mass to body weight (*P* < 0.0001). This result is consistent with the results of previous studies and the original function of subolesin, which indicates that vaccination has the same effectivity as RNAi treatment. In addition, the significant decrease in the egg mass to body weight ratio indicates that both are affected by vaccination, and the effect was more significant on egg mass. Interestingly, egg-hatching periods were extended significantly in the vaccinated group (*P* = 0.015). To the best of our knowledge, this study is the first to show that subolesin affects not only blood-feeding and egg mass but also the egg-hatching period. However, additional assessments are required to confirm that subolesin vaccination has the same function in other tick species.

This study evaluated blood-feeding periods because subolesin affects blood-feeding and may be affected by short blood-feeding periods. However, there was no difference between the two groups. Moreover, the pre-oviposition period was also not different.

Tick vaccines can control ticks by affecting different factors such as mortality, molting, number of infested ticks, fertility, and oviposition. Contreras et al. [26] showed that a higher mortality was observed in a subolesin-vaccinated group than in a control group; however, a difference in mortality was not observed in this study. In this study, some ticks abnormally detached after they started feeding; however, the number of detached ticks did not differ between the two groups. Since the model ticks were adults in this study, we focused on factors related to blood-feeding and reproduction. Additional studies are required to assess the effect of subolesin-based vaccination on the mortality of eggs and molting of *H. longicornis* in the larval and nymph stages.

In this study, the antibody levels reached their highest after the first vaccination and appeared to be sustained for more than two months (Fig. 3). Even after experimental infestation, the antibody levels did not increase further. Similar trends were identified after diluting the serum up to 1:80,000 (Additional file 1: Figure S1). According to previous studies, the effectiveness of vaccination is related to antibody levels against subolesin in the host [22, 35]. Based on the current vaccine formula, a one-time vaccination might be enough to obtain the objective antibody levels in the host. As *H. longicornis* is a three-host tick and has a longer life-cycle than *R. microplus*, which is a one-host tick, this vaccination can effectively control *H. longicornis* in endemic areas during the prevailing season, with a fewer number of vaccinations.

In this study, rHlSu was used for vaccination without removing the tags (His and ProS2 tag). Owing to the remaining tags, we cannot exclude the possibility of a non-specific immune response. However, based on the previous studies, subolesin accompanying different tags such as His-, Trx- and S-tag showed consistent vaccination results [22, 34]. Therefore, we suppose that even if there is a non-specific immune response, the response has minor effects on the experiment.

## Conclusions

Vaccination of rabbits with rHlSu significantly reduced body weight, egg mass, and egg mass to body weight ratio in *H. longicornis*. In addition, egg-hatching periods were also significantly extended. Finally, the vaccine efficacy was 37.4%. This finding raises the possibility that vaccination may gradually decrease the number of offspring. Variables related to the blood-feeding and pre-oviposition periods were not different between groups. In conclusion, this study showed the efficacy of rHlSu as a tick vaccine against *H. longicornis* in rabbits. To our knowledge, this is the first vaccination study of subolesin in *Haemaphysalis* spp., an important tick in Asian countries and an emerging tick in North America. Combined with the results of previous studies, this study suggests that subolesin has the potential to be used as a universal tick vaccine.

## Supplementary information

**Additional file 1: Table S1.** Effect of rHlSu vaccination on control of *H. longicornis* infestation in rabbits. **Figure S1.** Evaluation of antibody titers.

## Data Availability

The datasets supporting the conclusions of this article are included within the article and its additional files. The newly generated sequence was deposited in the GenBank database under the accession number MT199422.

## Abbreviations

ELISA: enzyme-linked immunosorbent assay
HlSu: *H. longicornis subolesin*
ORF: open reading frame
PBS: phosphate-buffered saline
PBS-T: PBS containing 0.1% Tween 20
PCR: polymerase chain reaction
rHlSu: recombinant HlSu
SDS-PAGE: sodium dodecyl sulfate polyacrylamide gel electrophoresis
SFTS: severe fever with thrombocytopenia syndrome
TBS-T: TBS containing 0.1% Tween 20

## References

[1] Jongejan F, Uilenberg G (2004). The global importance of ticks. Parasitology.

[2] Lee SH, Mossaad E, Ibrahim AM, Ismail AA, Moumouni PFA, Liu M (2018). Detection and molecular characterization of tick-borne pathogens infecting sheep and goats in Blue Nile and West Kordofan states in Sudan. Ticks Tick Borne Dis.

[3] Kim BJ, Kim H, Won S, Kim HC, Chong ST, Klein TA (2014). Ticks collected from wild and domestic animals and natural habitats in the Republic of Korea. Korean J Parasitol.

[4] Shimada Y, Beppu T, Inokuma H, Okuda M, Onishi T (2003). Ixodid tick species recovered from domestic dogs in Japan. Med Vet Entomol.

[5] Raghavan RK, Barker SC, Cobos ME, Barker D, Teo EJM, Foley DH (2019). Potential spatial distribution of the newly introduced long-horned tick, *Haemaphysalis longicornis* in North America. Sci Rep.

[6] Zhuang L, Sun Y, Cui XM, Tang F, Hu JG, Wang LY (2018). Transmission of severe fever with thrombocytopenia syndrome virus by *Haemaphysalis longicornis* ticks, China. Emerg Infect Dis.

[7] Higuchi S, Simomura S, Yoshida H, Hoshi F, Kawamura S, Yasuda Y (1991). Development of *Babesia gibsoni* in the hemolymph of the vector tick, *Haemaphysalis longicornis*. J Vet Med Sci.

[8] Hatta T, Matsubayashi M, Miyoshi T, Islam MK, Alim MA, Yamaji K (2013). Quantitative PCR-based parasite burden estimation of *Babesia gibsoni* in the vector tick, *Haemaphysalis longicornis* (Acari: Ixodidae), fed on an experimentally infected dog. J Vet Med Sci.

[9] George JE, Pound JM, Davey RB (2004). Chemical control of ticks on cattle and the resistance of these parasites to acaricides. Parasitology.

[10] Hayes EB (2010). Is it time for a new yellow fever vaccine?. Vaccine.

[11] Riley EM, Stewart VA (2013). Immune mechanisms in malaria: new insights in vaccine development. Nat Med.

[12] Nene V, Kiara H, Lacasta A, Pelle R, Svitek N, Steinaa L (2016). The biology of *Theileria parva* and control of East Coast fever—current status and future trends. Ticks Tick Borne Dis.

[13] Willadsen P, Riding GA, McKenna RV, Kemp DH, Tellam RL, Nielsen JN (1989). Immunologic control of a parasitic arthropod. Identification of a protective antigen from *Boophilus microplus*. J Immunol.

[14] de la Fuente J, Almazán C, Canales M, de la Lastra JMP, Kocan KM, Willadsen P (2007). A ten-year review of commercial vaccine performance for control of tick infestations on cattle. Anim Health Res Rev.

[15] Trentelman JJ, Teunissen H, Kleuskens JA, van de Crommert J, de la Fuente J, Hovius JW (2019). A combination of antibodies against Bm86 and Subolesin inhibits engorgement of *Rhipicephalus australis* (formerly *Rhipicephalus microplus*) larvae *in vitro*. Parasit Vectors.

[16] Coumou J, Wagemakers A, Trentelman JJ, Nijhof AM, Hovius JW (2015). Vaccination against Bm86 homologues in rabbits does not impair *Ixodes ricinus* feeding or oviposition. PLoS ONE.

[17] Rodríguez-Valle M, Taoufik A, Valdés M, Montero C, Hassan I, Hassan SM (2012). Efficacy of *Rhipicephalus* (*Boophilus*) *microplus* Bm86 against *Hyalomma dromedarii* and *Amblyomma cajennense* tick infestations in camels and cattle. Vaccine.

[18] Liao M, Zhou J, Hatta T, Umemiya R, Miyoshi T, Tsuji N (2007). Molecular characterization of *Rhipicephalus* (*Boophilus*) *microplus* Bm86 homologue from *Haemaphysalis longicornis* ticks. Vet Parasitol.

[19] Almazán C, Kocan KM, Bergman DK, Garcia-Garcia JC, Blouin EF, de la Fuente J (2003). Identification of protective antigens for the control of *Ixodes scapularis* infestations using cDNA expression library immunization. Vaccine.

[20] de la Fuente J, Almazán C, Blas-Machado U, Naranjo V, Mangold AJ, Blouin EF (2006). The tick protective antigen, 4D8, is a conserved protein involved in modulation of tick blood ingestion and reproduction. Vaccine.

[21] Galindo RC, Doncel-Perez E, Zivkovic Z, Naranjo V, Gortazar C, Mangold AJ (2009). Tick subolesin is an ortholog of the akirins described in insects and vertebrates. Dev Comp Immunol.

[22] Moreno-Cid JA, de la Lastra JMP, Villar M, Jiménez M, Pinal R, Estrada-Peña A (2013). Control of multiple arthropod vector infestations with subolesin/akirin vaccines. Vaccine.

[23] Merino O, Almazán C, Canales M, Villar M, Moreno-Cid JA, Galindo RC (2011). Targeting the tick protective antigen subolesin reduces vector infestations and pathogen infection by *Anaplasma marginale* and *Babesia bigemina*. Vaccine.

[24] Zivkovic Z, Torina A, Mitra R, Alongi A, Scimeca S, Kocan KM (2010). Subolesin expression in response to pathogen infection in ticks. BMC Immunol.

[25] Bensaci M, Bhattacharya D, Clark R, Hu LT (2012). Oral vaccination with vaccinia virus expressing the tick antigen subolesin inhibits tick feeding and transmission of *Borrelia burgdorferi*. Vaccine.

[26] Contreras M, de la Fuente J (2016). Control of *Ixodes ricinus* and *Dermacentor reticulatus* tick infestations in rabbits vaccinated with the Q38 Subolesin/Akirin chimera. Vaccine.

[27] Rahman M, Saiful Islam M, You M (2018). Impact of subolesin and cystatin knockdown by RNA interference in adult female *Haemaphysalis longicornis* (Acari: Ixodidae) on blood engorgement and reproduction. Insects.

[28] Almazán C, Lagunes R, Villar M, Canales M, Rosario-Cruz R, Jongejan F (2010). Identification and characterization of *Rhipicephalus* (*Boophilus*) *microplus* candidate protective antigens for the control of cattle tick infestations. Parasitol Res.

[29] Nijhof AM, Taoufik A, De la Fuente J, Kocan KM, De Vries E, Jongejan F (2007). Gene silencing of the tick protective antigens, Bm86, Bm91 and subolesin, in the one-host tick *Boophilus microplus* by RNA interference. Int J Parasitol.

[30] Umemiya-Shirafuji R, Fujisaki K, Okado K, Moumouni PFA, Yokoyama N, Suzuki H (2019). Hard ticks as research resources for vector biology: from genome to whole-body level. Med Entomol Zool.

[31] Umemiya-Shirafuji R, Matsuo T, Liao M, Boldbaatar D, Battur B, Suzuki HI (2010). Increased expression of ATG genes during nonfeeding periods in the tick *Haemaphysalis longicornis*. Autophagy.

[32] Kamio T (1987). The improvemetn of “ear bag” method for tick infestation. Proc Jpn Assoc Acarol..

[33] Prudencio CR, de la Lastra JMP, Canales M, Villar M, de la Fuente J (2010). Mapping protective epitopes in the tick and mosquito subolesin ortholog proteins. Vaccine.

[34] Shakya M, Kumar B, Nagar G, de la Fuente J, Ghosh S (2014). Subolesin: A candidate vaccine antigen for the control of cattle tick infestations in Indian situation. Vaccine.

[35] Merino O, Antunes S, Mosqueda J, Moreno-Cid JA, de la Lastra JMP, Rosario-Cruz R (2013). Vaccination with proteins involved in tick-pathogen interactions reduces vector infestations and pathogen infection. Vaccine.

