# Supplementary material for: Subolesin vaccination inhibits blood feeding and reproduction of Haemaphysalis longicornis in rabbits
Source: Parasit Vectors. 2020 Sep 18;13:478. doi: 10.1186/s13071-020-04359-w (PMC7501621; doi:10.1186/s13071-020-04359-w)
Supplement: Supplementary file 1 — Additional file 1: Table S1. Effect of rHlSu vaccination on control of H. longicornis infestation in rabbits. Figure S1. Evaluation of antibody titers. [file 13071_2020_4359_MOESM1_ESM.docx]

**Additional file 1: Table S1.** Effect of rHlSu vaccination on control of *H. longicornis* infestation in rabbits

| Group | Rabbit No. | No. of engorged ticks | Blood feeding period (days) | Pre-oviposition period  (days) | Body weight at engorgement (mg) | Egg mass weight at 10 days after oviposition (mg) | Egg mass to body weight ratio | Egg hatching period (days) |
| --- | --- | --- | --- | --- | --- | --- | --- | --- |
| Vaccinated | #2 | 40 | 7.4 | 5.8 | 217.3 | 78.1 | 36.9 | 35.9 |
|  | #3 | 37 | 8.8 | 7.2 | 190.5 | 44.0 | 23.1 | 41.1 |
|  | #4 | 38 | 6.8 | 6.5 | 192.2 | 62.0 | 32.0 | 38.7 |
|  | Average values | 115 | 7.7±1.24 | 6.5±1.12 | 200.4±44.13 | 61.8±21.94 | 30.9±8.97 | 38.4±3.74 |
| PBS | #5 | 39 | 7.9 | 5.4 | 244.6 | 110.0 | 44.5 | 37.1 |
|  | #6 | 36 | 7.5 | 6.7 | 220.2 | 84.1 | 37.5 | 35.0 |
|  | #7 | 38 | 7.1 | 7.2 | 212.2 | 65.7 | 31.0 | 39.3 |
|  | Average values | 114 | 7.5±0.93 | 6.4±1.11 | 227.6±52.65 | 86.9±30.20 | 37.7±8.65 | 37.1±3.86 |
| *p*-value |  |  | 0.221 | 0.676 | <0.0001 | <0.0001 | <0.0001 | 0.015 |

Results are shown for each rabbit. The average values are described with the standard deviation. Average values between the groups were analyzed by the Student’s t-test or Mann-Whitney test.

**Additional file 1: Figure S1.** Evaluation of antibody titers. Results are shown as average values in the vaccination group. Antibody levels were estimated using ELISA. Following each vaccination, there was a two-week interval until the antibody levels were assessed.

**
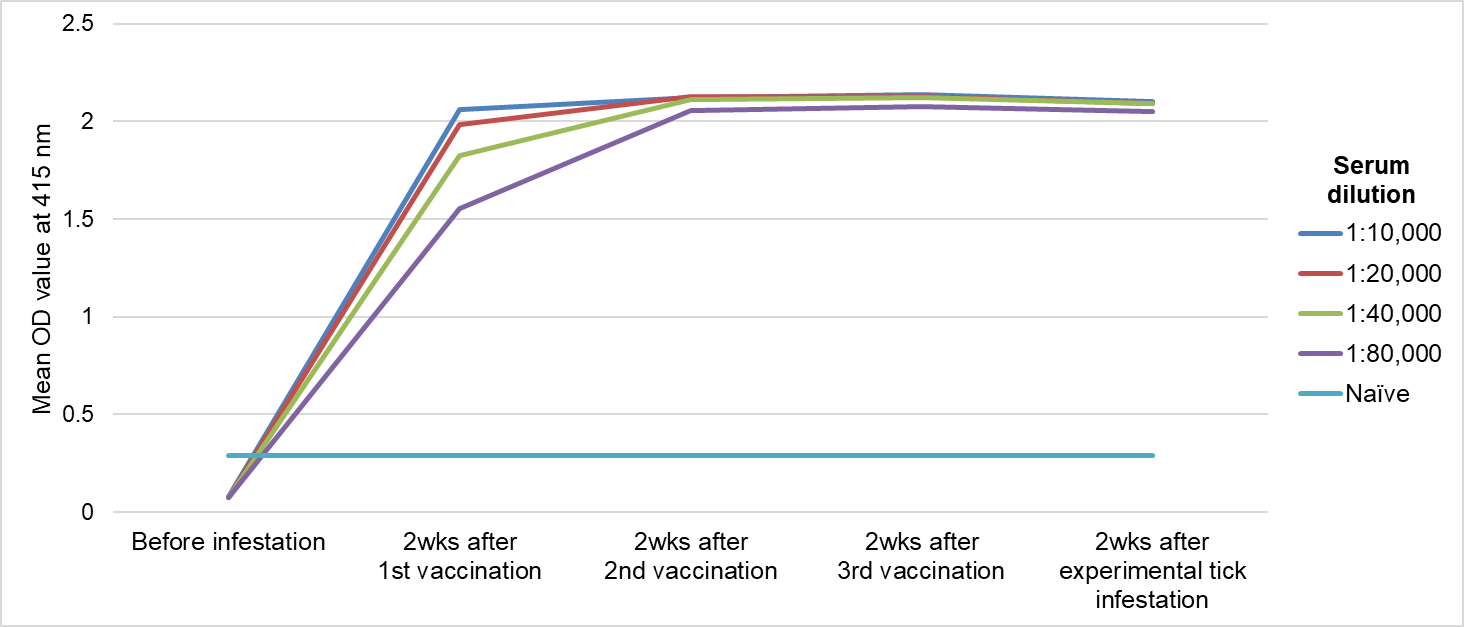
**
